# Medical Complications Among Children and Adolescents with Sickle Cell Disease in Texas Medicaid

**DOI:** 10.3390/healthcare13182288

**Published:** 2025-09-12

**Authors:** Gloria N. Odonkor, Hyeun Ah Kang, Jamie C. Barner, Kenneth A. Lawson, Titilope Fasipe

**Affiliations:** 1 Health Outcomes Division, College of Pharmacy, The University of Texas at Austin, Austin, TX 78712, USA; g.odonkor@utexas.edu (G.N.O.); jbarner@austin.utexas.edu (J.C.B.); ken.lawson@austin.utexas.edu (K.A.L.); 2 Division of Hematology/Oncology, Department of Pediatrics, Baylor College of Medicine, Texas Children’s Hospital, Houston, TX 77030, USA; taishola@texaschildrens.org

**Keywords:** complications, children, adolescents, sickle cell disease

## Abstract

**Highlights:**

**What are the main findings?**

- Younger children had a higher number of documented complications and affected organ systems compared to the older age group, highlighting the need for early preventive measures and screening for organ damage.
- In contrast to the nature of disease progression, the documented number of complications was lower in adolescents (aged 15–18) compared to younger children (aged 2–14), underscoring the need for improved healthcare access among this age group to ensure continuity of care as well as a better transition to adult care.

**What is the implication of the main finding?**

- The above findings demonstrate the need for improvement in the utilization of preventive therapy in younger children, and for better access to healthcare among adolescents with SCD.
- The high prevalence of mental health comorbidities (anxiety and/or depression) and their association with experiencing the most salient SCD complications requires attention to early monitoring and intervention for mental health comorbidities in this population.
- Hydroxyurea was underutilized among children and adolescents with SCD, indicating a critical gap in treatment that warrants targeted interventions.

**Abstract:**

**Background**: Patients with sickle cell disease (SCD) experience severe and life-threatening complications over their lifespans. However, research on SCD age-related complications is limited. **Objective**: This study examined differences in the number and type of SCD-related complications by age group among Texas Medicaid pediatric patients, and the factors associated with salient complications. **Methods**: This retrospective study used Texas Medicaid prescription and medical claims (2012–2021). Subjects aged 2 to 18 years, with ≥3 SCD hospitalizations or outpatient visits, and continuously enrolled for ≥12 months after the first SCD diagnosis claim were included. Complications were characterized by number and type of organ systems affected. Sociodemographic and clinical factors were used as potential factors associated with the most salient complications. Descriptive and inferential (ANOVA, Chi-square, and multivariable logistic regression) analyses were employed. **Results**: The included 1555 patients (mean age = 9.5 ± 5.1) were categorized into four age groups: 2–4 (23.4%), 5–9 (26.9%), 10–14 (27.4%), and 15–18 (22.3%) years. Documented number and type of complications differed significantly (all *p* < 0.0001) by age group, with the 2–14 years group having more documented complications compared to the 15–18 years group. Neurological complications were most common (~65%), followed by infections (~42%), and cardio-pulmonary complications (~30%). Young age group, hydroxyurea use, and having mental health comorbidities were associated with greater likelihood of experiencing vaso-occlusive crises, respiratory infections, and acute chest syndrome. **Conclusions**: Patterns of SCD-related complications (e.g., VOCs, respiratory infections, and acute chest syndrome) differ significantly by age group, leading to increased morbidity and acute care utilization. Despite its reported association with better outcomes, hydroxyurea utilization was found to be poor, with only 16% of patients receiving it for at least 180 days annually. Access to appropriate healthcare and improved utilization of hydroxyurea are needed to improve health outcomes of this population over their lifespan.

## 1. Introduction

Sickle cell disease (SCD) is one of the most common and severe monogenic disorders in the world [1]. In the United States (US), about 100,000 people live with SCD, approximately 90% of whom are Black and 10% Hispanic [2]. Every year, SCD is identified in about 2000 infants in the US, with an estimated 1 in 365 African American births and 1 in 16,300 Hispanic American births [3]. Complex pathophysiological processes consisting of vaso-occlusion, inflammation, and tissue ischemia [4] cause a wide range of acute complications such as vaso-occlusive crisis (VOC), acute chest syndrome (ACS), and stroke in patients with SCD. Recurring vaso-occlusion, hemolysis, and ischemia lead to chronic complications such as chronic pain and multi-organ damage [4]. The clinical complications of SCD vary by age [5]. The disease is asymptomatic in the first 1 to 2 months of life, but beyond that, complications such as dactylitis and splenic dysfunction emerge. In childhood, acute splenic sequestration, infections, stroke, and ACS are common [5]; and in adolescence, patients may begin to suffer from complications such as leg ulcers, priapism, and avascular necrosis [5]. In adulthood, many of these complications persist, and problems such as pulmonary hypertension, renal disease, and cardiovascular complications become major contributors to multisystem organ damage [5].

Studies show that among children with SCD, hospitalizations are approximately 7–30 times higher than for same-age cohorts without SCD [6,7]. Health resource utilization is even higher among adolescents and young adults (AYAs) who are transitioning from pediatric to adult care, and they have the highest rates of pain crises, hospitalizations, and re-hospitalizations [7,8,9,10,11]. Although these poor outcomes in children and AYAs with SCD may be due to age-related changes in complications and potential care disruptions at critical stages in the lifespan of patients with this disease, there is not much information on the age of onset and patterns of SCD complications that may be contributing to these adverse health outcomes.

Because of the variability and complexity of the many manifestations of SCD, information on the age-related trends in the symptoms and complications of SCD has been recognized as an essential component in the development of programs to facilitate the management and potential prevention of these complications. However, data on this is limited and mostly outdated [5,12]. Most studies have focused on SCD-related healthcare utilization (i.e., hospitalizations, re-hospitalizations, and emergency department visits) in children and AYAs [7,8,10,11,13], with little real-world data on the complications of SCD in this population that impact these suboptimal health outcomes.

This study presents a broader view of the shift in the clinical burden of SCD by describing the age-related patterns in SCD complications and factors that influence the occurrence of these complications. Specifically, this study aims to determine (1) if there are differences in the number and type of medical complications by age groups, and (2) whether factors such as age group, sex, resource availability, hydroxyurea use, and mental health comorbidities are associated with the most salient clinical complications among children and adolescents with SCD enrolled in Texas Medicaid.

## 2. Methods

### 2.1. Study Design and Data Source

This was a retrospective secondary database analysis using Texas Medicaid claims data of patients with SCD from March 2012 through May 2021 (study period). Texas has the third-largest population of patients with SCD [2]. Additionally, among Medicaid and CHIP beneficiaries (50–60% of SCD patients), Texas is ranked fourth among the 50 states within the US, making it important to better understand the disease burden of this population [14]. The data included de-identified unique patient numbers, eligibility information, medical outpatient and inpatient claims, and pharmacy claims.

### 2.2. Patient Population

Texas Medicaid beneficiaries were included in the study if they were aged between 2 and 18 years at index date (the date of the first observed claim with a diagnosis of SCD between March 2012 and May 2020 [identification period]) and were continuously enrolled in Texas Medicaid for at least 12 months after the index date (defined as having one or more SCD-related medical or prescription claims in at least 3 out of the 4 quarters of the year). Additionally, to validly identify SCD in real world data, those who had at least 3 hospitalizations or outpatient visits associated with a diagnosis of SCD (International Classification of Diseases, Ninth/Tenth Revision, Clinical Modification [ICD-9-CM] codes: 282.41, 282.42, 282.60–282.69 or [ICD-10-CM] codes: D57, D57.x and D57.xx, excluding D57.3 [sickle cell trait]) [15] during the study period were included.

### 2.3. Study Variables

The key outcomes measured in this study included the number and type of documented medical complications. In consultation with a pediatric clinical hematologist who treats SCD (TF), type of complications was grouped into organ-specific complications namely cardiovascular, cardio-pulmonary, cerebrovascular, dermatological, gastrointestinal, genitourinary, hematological, musculoskeletal, neurological (pain), ophthalmologic, pulmonary, renal, splenic, and other (infection and severe infection) complications, using ICD-9 and ICD-10 codes [Supplementary Table S1]. In the multivariable logistic regression, the dependent variable was having at least one of the most salient medical complications, defined as those that occurred in more than five percent of the study sample. Although there is no established threshold for defining salient complications, five percent was chosen as the cut-off for clinical relevance in this study, based on the distribution of the frequency of complications in our sample (several complications were very low-frequency events). The primary independent variable was age group (i.e., 2–4 years, 5–9 years, 10–14 years, and 15–18 years). Other covariates included sex, resource availability, use of hydroxyurea, and mental health comorbidities. Resource availability was defined as the presence of SCD resources (i.e., SCD/specialty health centers) within a 50-mile radius from a patient’s residence [16]; hydroxyurea use was defined as ≥180 days’ supply of hydroxyurea [17]; and mental health comorbidities were defined as having a diagnosis of anxiety and/or depression during the study period.

### 2.4. Statistical Analyses

Means (standard deviation; SD) or frequencies (percentage) were used to describe continuous and categorical variables, respectively. Analysis of variance (ANOVA) and Pearson one-sample chi-square tests were used to evaluate continuous and categorical variables, respectively, to determine whether there were significant differences in the number and type of documented complications experienced by age group. Furthermore, multivariable logistic regression analyses were conducted to evaluate which factors, including age group and the above-mentioned covariates, were associated with having at least one of the most salient medical complications. All statistical tests were conducted using SAS version 9.4 software (SAS Institute, Cary, NC, USA). A significance level of *p* < 0.05 was used for all analyses unless otherwise indicated.

## 3. Results

### 3.1. Sample Characteristics

**Figure 1:**
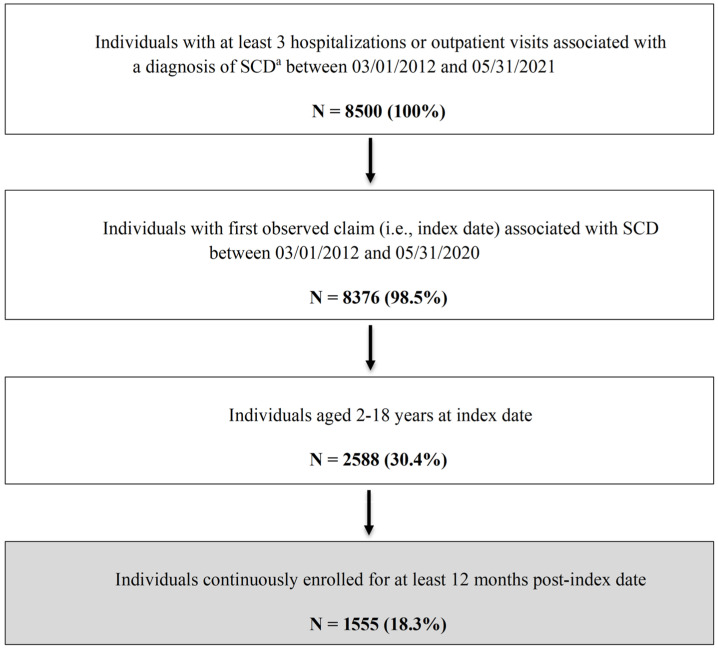
Flowchart of Patient Selection Process. ^a^ ICD-9-CM codes 282.41, 282.42, and 282.60–282.69 or ICD-10-CM codes D57, D57.x and D57.xx, excluding D57.3 (sickle cell trait). ICD-9/10-CM = International Classification of Diseases, Ninth/Tenth Revision, Clinical Modification; SCD = sickle cell disease.

A total of 1555 patients with SCD were included in this study [Figure 1]. Table 1 summarizes the key patient characteristics. The overall mean age was 9.5 ± 5.1 years. While fairly evenly distributed, those aged 10–14 years accounted for the highest proportion of the study sample (27.4%), followed by those aged 5–9 years (26.9%). Slightly more than half of the sample were female (55.3%). The majority of the study population (80.9%) lived in an area with an SCD resource within 50 miles of their residence, only 16% of patients were on hydroxyurea for ≥180 days, and 33.3% had at least one mental health (anxiety and/or depression) diagnosis.

### 3.2. Number and Frequency of Medical Complications by Age Group

**Figure 2:**
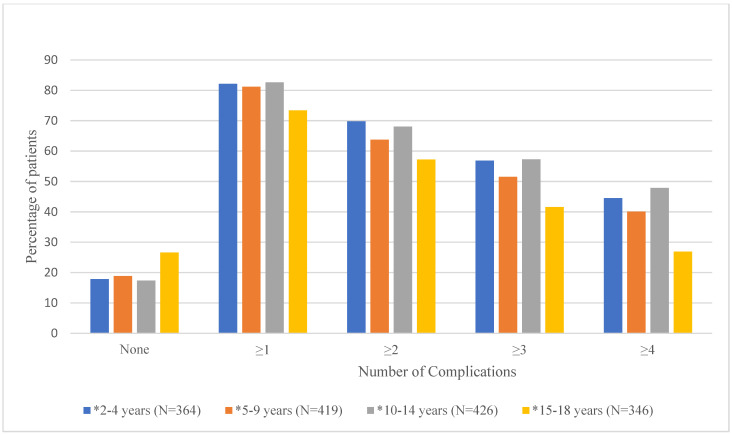
Proportion of Patients with 0, ≥1, ≥2, ≥3, or ≥4 Documented Complications by Age Group. * denotes significant *p*-values (<0.05) for chi-square tests.

Mean number of documented (i.e., having an ICD-9 or ICD-10 code in the database) complications and the proportion of patients experiencing 0, ≥1, ≥2, ≥3, and ≥4 complications by age group are shown in Table 2 and Figure 2, respectively. As shown in Table 2, the mean number of documented complications ranged from 2.4 ± 2.2 for the 15–18-year age group to 3.9 ± 3.4 for the 2–4-year age group. Results from the ANOVA test showed that overall, the mean number of documented complications differed significantly by age group (*p* < 0.0001). Duncan’s post hoc analyses showed that age groups 2–4 (3.9 ± 3.4), 5–9 (3.5 ± 3.3), and 10–14 (3.7 ± 3.3) had significantly higher mean number of documented complications than the 15–18 (2.4 ± 2.2) age group. Figure 2 shows that there are significant differences in the proportion of patients with 0, ≥1, ≥2, ≥3, or ≥4 documented complications for each age group [Supplementary Table S2].

### 3.3. Type of Medical Complications (Organ System Involvement) by Age Group

**Figure 3:**
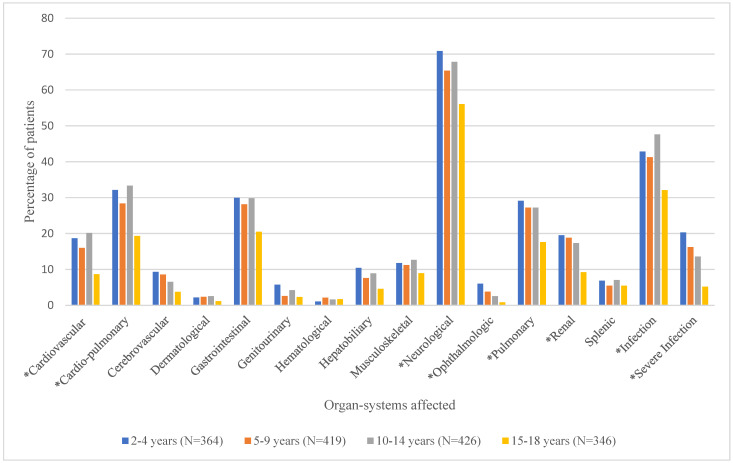
Proportion of Patients with Various Types of Documented Organ-System Complications by Age Group. * denotes significant *p*-values (<0.05) for chi-square tests.

Mean number of organ systems affected and the proportion of patients experiencing the various organ-system complications by age group are shown in Table 2 and Figure 3, respectively. As shown in Table 2, the mean number of documented organ-system complications ranged from 2.0 ± 1.7 for the 15–18-year age group to 3.2 ± 2.6 for the 2–4-year age group. ANOVA test results showed that overall, the mean number of organ-system complications differed significantly by age group (*p* < 0.0001). Duncan’s post hoc comparisons revealed that age groups 2–4 (3.2 ± 2.6), 5–9 (2.9 ± 2.5), and 10–14 (3.0 ± 2.4), had significantly higher numbers of documented organ-system complications than the 15–18 (2.0 ± 1.7) age group. Overall, neurological complications (e.g., VOC) were the most common among this population, followed by infection and cardio-pulmonary complications. Figure 3 shows that there were significant differences in the proportion of patients with cardiovascular, cardio-pulmonary, neurological (pain), ophthalmologic, pulmonary, renal, and other (infection and severe infections) complications by age group [Supplementary Table S3].

### 3.4. Factors Associated with Experiencing at Least One of the Most Salient Medical Complications

Salient documented medical complications by frequency were VOCs (18.9%), respiratory infections (9.1%), constipation (8.1%), asthma (7.5%), fever (7.4%), ACS (7.3%), and dactylitis (6.5%). Regression results indicated that the age group was significantly associated with the odds of experiencing at least one of the most salient medical complications after controlling for covariates. There was a 30.9% significantly lower odds of experiencing at least one of the most salient complications among patients aged 15–18 years compared to those aged 2–4 years (OR = 0.691, 95% CI = 0.483–0.991, *p* = 0.0442). None of the other age group categories showed a significant difference in the likelihood of experiencing at least one of the most salient complications compared to the reference group (2–4 years). Patients who were on hydroxyurea for 180 days or longer had 10.8 times higher odds of experiencing at least one of the most salient complications compared to those who were not (OR = 10.833, 95% CI = 5.292–22.173, *p* < 0.0001). To provide more complication-specific information, individual models were run for the top three salient complications selected using frequency and clinical relevance (VOCs, respiratory infections, and ACS). For these models, in addition to age group and hydroxyurea use, patients with mental health comorbidities had significantly higher odds of experiencing VOCs and ACS compared to those who did not have these comorbidities [Supplementary Table S4].

## 4. Discussion

Knowledge of the clinical progression of disease has been recognized as a critical component in the advancement of interventions for conditions such as SCD, which can be identified early through newborn screening. Limited literature on the natural history of SCD shows the trends in the onset of complications at various stages of life, with many complications that are identified in childhood persisting till adulthood, leading to poor health outcomes [5,6,18,19]. Results from this study of age-related medical complications in children with SCD show that there were significant differences in the occurrence of complications by age group. Contrary to what has been observed in patients and previous studies that reported an increase in the mean number of complications per patient after age 15 [10,12,20,21,22], our results showed a decrease in the documentation of diagnosed medical complications in those aged 15–18 years. These findings should be interpreted in the context of the nature of disease progression of SCD, as well as potential access limitations. After the first few months of the asymptomatic period, complications such as dactylitis, splenic dysfunction, infections, stroke, and ACS typically emerge during childhood, and additional complications such as leg ulcers, priapism, and avascular necrosis tend to develop in adolescence [5]. Therefore, rather than reflecting a true reduction in the number of complications, the observed decrease in the older age group may be due to barriers to accessing care, which could lead to a reduction in routine visits and follow-ups with primary or specialty healthcare providers, and could potentially lead to the underdiagnosis/underreporting of complications among this age group [23,24,25,26,27,28]. In a Texas Medicaid study of SCD beneficiaries that examined age-related healthcare services utilization, the authors found that outpatient visits were significantly lower for ages 13–17 compared to those 2–12 (2.9 ± 5.4 vs. 4.5 ± 7.6, respectively) [28], which may also indicate access to care barriers in older age groups (i.e., the 15–18 age group in the current study). Furthermore, it is important to note that because of the cross-sectional nature of our study, the numbers reported do not reflect the lifetime accumulated complications of individuals and thus should be interpreted with caution.

Research suggests that the organ systems affected by SCD complications differ by age [29,30], as was observed in this study. Because of the variability in the clinical manifestations of SCD, there is a growing interest in identifying patients at high risk of developing long-term outcomes related to organ dysfunction at the earliest possible age to potentially prevent associated negative health consequences. In the current study, patients aged 2–4 years had more than three (3.2 ± 2.6) organ systems affected by SCD-related complications, on average, with neurological (pain), infection, and cardio-pulmonary systems being the most prevalent three organ systems impacted by complications among this age group. Neurological complications explored in this study included VOCs and chronic pain syndrome; cardio-pulmonary complications included ACS, thromboembolism, and pulmonary hypertension; infections included fever and upper respiratory tract infections. Although recommended therapies are available to help reduce the occurrence of complications of SCD, relatively few patients receive these medications. According to the CDC, only 2 in 5 children (38%) aged 2–9 years used hydroxyurea to prevent SCD-related complications in 2019 [31]. Our study also showed that only 16% of those aged 2–18 were on hydroxyurea for at least 180 days in the year, confirming the underutilization of this guideline-recommended therapy [32] in this population. This underutilization may explain our observations of the higher mean numbers of organ systems affected for those aged 2–14 years in our sample and highlight the barriers and challenges that children and their families face in receiving appropriate care within the healthcare system. While our study did not explore dosing patterns or adherence to hydroxyurea, it is possible that differing levels of real-world adherence to the medication may have an impact on complication rates and other health outcomes. Similarly to the mean number of complications, the lower numbers of documented organ systems affected by complications for those aged 15–18 years may be due to the lack of access to routine and specialty care in this population and the potential underreporting of complications, which may be clinically silent. In childhood, SCD is characterized by acute episodes of illness, which may lead to the diagnosis of organ-system complications, but from which children usually recover. In adolescence and adulthood, these organ-system complications may be less apparent and misdiagnosed or not diagnosed until significant organ damage occurs [18].

In terms of the factors associated with having at least one of the most salient medical complications defined in this study, those aged 15–18 years were less likely to experience these complications compared with the reference group of 2–4-year-olds, which is consistent with the trend identified in this study. Possible reasons, as indicated above, may explain this finding, particularly because these salient complications occur at different stages of an individual’s life. That is, rather than reflecting an actual protective effect in those aged 15–18 years, the observed decrease in the odds of experiencing the salient medical complications in the older age group may be due to barriers to accessing care, which could lead to reduction in routine visits and follow-ups with primary or specialty healthcare providers, and could potentially lead to the underdiagnosis/underreporting of complications among this age group. Resource availability was not significantly associated with the odds of experiencing at least one of the most salient medical complications. Nevertheless, there is a need for more comprehensive sickle cell centers, which can improve access to healthcare services, transition from pediatric to adult care, and long-term outcomes for patients with SCD.

Patients on hydroxyurea for 180 days or longer in a year had a higher likelihood of experiencing at least one of the most salient medical complications compared to those who did not. This result was similar to that of a study by Tripathi et al. [33], which showed that the use of hydroxyurea may have been targeted towards patients with more severe disease and higher rates of complications such as VOC and ACS, and not necessarily used as a preventive measure to decrease long-term complications and organ damage. However, the cross-sectional nature of this study does not allow for causality to be inferred from these results. Mental health comorbidities (anxiety and/or depression) were identified in 33.3% of our study sample, which is approximately 3 times higher than in the non-SCD population (i.e., 8.4% for children and adolescents [34] and 9.5% for adults [35,36]). Additionally, those with mental health conditions in this study were more likely to experience VOCs and ACS compared to those without, which is similar to results from another study [37]. As stated above, although the cross-sectional nature of the study does not allow for causality to be inferred, these findings indicate a dire need for increased screening and evaluation of patients with SCD as early as childhood for mental health comorbidities and the provision of early interventions for better disease management in this population. Longitudinal studies are needed to explore these relationships further. Future studies also need to be conducted to identify challenges in adolescents as they prepare to transition to adult care to inform the design of programs that support patients and improve their health outcomes.

This study has several limitations. First, because this study used diagnosis codes for the determination of disease complications and comorbidities, the actual rate of complications may be underestimated since only the most salient issues are typically coded in the patient’s medical record and complications managed at home were not captured. Also, due to limitations in data, outpatient claims could not be subclassified as preventative versus sick visits. The validity of the results may also depend on the accuracy of the coding. Second, since the Medicaid database for only one state was used, the results of this study may not be representative of patients with SCD in other states and the overall US Medicaid population. However, Texas has the third largest number of patients with SCD in the US. Third, because of the inclusion criterion of at least three hospitalizations or outpatient visits associated with an SCD diagnosis used in this study, patients included may have higher disease severity than the average SCD population. However, this is the definition that is recommended for use to enhance the validity of the diagnosis when analyzing Medicaid or Medicare data [15]. Fourth, due to the lack of laboratory or genetic data in the claims data, examination of the impact of disease genotype on the rates and types of complications was not possible. Fifth, the cross-sectional nature of the study does not allow for the determination of causality, especially for the relationship between receiving hydroxyurea therapy and experiencing salient medical complications. Lastly, it is difficult to control for the proximity of available SCD resources because zip codes may be misleading in identifying residents who live close to sickle cell resources.

## 5. Conclusions

Overall, this study addressed an important gap in knowledge and showed varying patterns in the prevalence of complications in children and adolescents with SCD, particularly for conditions that may be underreported in children, such as organ-system complications and comorbid mental health conditions. Contrary to the natural progression of disease, the documented number of complications was lower in adolescents (aged 15–18) compared to younger children (aged 2–14), highlighting the need for improved healthcare access among this age group to ensure continuity of care as well as a better transition to adult care. The high number of documented complications and affected organ systems in young children also underscores the need for early preventive measures and screening for organ damage. Underutilization of hydroxyurea showed the importance of early initiation and active adoption of the guideline-recommended therapy in this population. Further studies are needed to identify barriers to healthcare access in adolescents with SCD prior to the transition period, as well as barriers to the use of recommended hydroxyurea, to support the development of interventions that can improve the health outcomes of these patients.

## Figures and Tables

**Table 1:** Demographic Characteristics, Resource Availability, Hydroxyurea Use, and Mental Health Comorbidities of Study Population (N = 1555).

Characteristics	Patient Sample (N = 1555)	
Age at Index Date	Mean	SD
	9.5	5.1
Age Groups (years)	N	%
2–4	364	23.4
5–9	419	26.9
10–14	426	27.4
15–18	346	22.3
Gender	N	%
Male	695	44.7
Female	860	55.3
Resource Availability	N	%
Lives in area with SCD resource (<50 miles) ^a^	1258	80.9
Hydroxyurea use	N	%
Hydroxyurea use (≥180 days’ supply)	249	16.0
Hydroxyurea use (≥300 days’ supply) sensitivity analysis	179	11.5
Mental Health Comorbidities	N	%
Anxiety and/or depression diagnosis	518	33.3

SD = Standard Deviation. SCD = Sickle Cell Disease. ^a^ SCD resource = SCD/Specialty health centers.

**Table 2:** Comparison of Mean Number and Type (Organ Systems Affected) of Documented Complications by Age Group (N = 1555).

Age Group		Number of Complications	Number of Organ-Systems Affected
Category (Years)	N	Mean (SD)	Mean (SD)
2–4	364	3.9 (3.4) ^a^	3.2 (2.6) ^a^
5–9	419	3.5 (3.3) ^a^	2.9 (2.5) ^a^
10–14	426	3.7 (3.3) ^a^	3.0 (2.4) ^a^
15–18	346	2.4 (2.2) ^b^	2.0 (1.7) ^b^

ANOVA (Number of complications): F = 18.07; df = 3, 1551; *p* < 0.0001. ANOVA (Number of organ systems affected): F = 18.45; df = 3, 1551; *p* < 0.0001. ANOVA = Analysis of Variance. ^a,b^ Duncan’s post hoc: Like letters are not significantly different between age groups.

## Data Availability

Restrictions apply to the datasets: the datasets presented in this article are not readily available due to privacy protections under the Data Use Agreement between Texas Health and Human Services and The University of Texas at Austin College of Pharmacy Health Outcomes Division.

## Supplementary Materials

The following supporting information can be downloaded at https://www.mdpi.com/article/10.3390/healthcare13182288/s1: Table S1. ICD-9 and ICD-10 Codes for Medical Complications in Patients with Sickle Cell Disease. Table S2. Proportion of Patients with 0, ≥1, ≥2, ≥3, or ≥4 Documented Complications for Each Age Group (N=1555). Table S3. Proportion of Patients with Various Types of Documented Organ-System Complications by Age Group (N=1555). Table S4. Logistic Regression Analysis Examining the Likelihood of Experiencing Salient Documented Medical Complications by Age Group.

## References

[1] Rees D.C., Williams T.N., Gladwin M.T. (2010). Sickle-cell disease. Lancet.

[2] Hassell K.L. (2010). Population estimates of sickle cell disease in the U.S. Am. J. Prev. Med..

[3] Therrell B.L., Lloyd-Puryear M.A., Eckman J.R., Mann M.Y. (2015). Newborn Screening for Sickle Cell Diseases in the united States: A Review of Data Spanning 2 Decades. Semin. Perinatol..

[4] Ware R.E., de Montalembert M., Tshilolo L., Abboud M.R. (2017). Sickle cell disease. Lancet.

[5] Serjeant G.R. (2013). The natural history of sickle cell disease. Cold Spring Harb. Perspect. Med..

[6] Tanabe P., Spratling R., Smith D., Grissom P., Hulihan M. (2019). CE: Understanding the complications of sickle cell disease. Am. J. Nurs..

[7] Shankar S.M., Arbogast P.G., Mitchel E., Cooper W.O., Wang W.C., Griffin M.R. (2005). Medical care utilization and mortality in sickle cell disease: A population-based study. Am. J. Hematol..

[8] Brousseau D.C., Owens P.L., Mosso A.L., Panepinto J.A., Steiner C.A. (2010). Acute care utilization and rehospitalizations for sickle cell disease. JAMA.

[9] Lebensburger J.D., Bemrich-Stolz C.J., Howard T.H. (2012). Barriers in transition from pediatrics to adult medicine in sickle cell anemia. J. Blood Med..

[10] Blinder M.A., Duh M.S., Sasane M., Trahey A., Paley C., Vekeman F. (2015). Age-related emergency department reliance in patients with sickle cell disease. J. Emerg. Med..

[11] Dickerson A.K., Klima J., Rhodes M.M., O’Brien S.H. (2012). Young adults with SCD in US children’s hospitals: Are they different from adolescents?. Pediatr. Blood Cancer.

[12] Blinder M.A., Vekeman F., Sasane M., Trahey A., Paley C., Duh M.S. (2013). Age-related treatment patterns in sickle cell disease patients and the associated sickle cell complications and healthcare costs. Pediatr. Blood Cancer.

[13] Dampier C., Ely B., Bs D.B., Coleman C., Aertker L., Sendecki J.A., Leiby B., Kesler K., Hyslop T., Stuart M. (2014). Pain characteristics and age-related pain trajectories in infants and young children with sickle cell disease. Pediatr. Blood Cancer.

[14] Wilson-Frederick S., Hulihan M., Mangum A., Khan T., Geibel M., Malsberger R., Verghese R., Borck R., Fox R., Rosenbach M. (2021). Medicaid and CHIP Sickle Cell Disease Report, T-MSIS Analytic Files (TAF) 2017.

[15] Chronic Conditions Warehouse Other Chronic Health, Mental Health, and Potentially Disabling Conditions—Sickle Cell Disease. MBSF_OTCC_{YYYY} File. https://www2.ccwdata.org/web/guest/condition-categories-other.

[16] Kayle M., Docherty S.L., Sloane R., Tanabe P., Maslow G., Pan W., Shah N. (2019). Transition to adult care in sickle cell disease: A longitudinal study of clinical characteristics and disease severity. Pediatr. Blood Cancer.

[17] Reeves S.L., Jary H.K., Gondhi J.P., Raphael J.L., Lisabeth L.D., Dombkowski K.J. (2019). Hydroxyurea use among children with sickle cell anemia. Pediatr. Blood Cancer.

[18] Pecker L.H., Little J. (2017). Clinical manifestations of sickle cell disease across the lifespan. Sickle Cell Disease and Hematopoietic Stem Cell Transplantation.

[19] Vichinsky E.P. (2014). Overview of the clinical manifestations of sickle cell disease. Pain.

[20] Paulukonis S.T., Feuchtbaum L.B., Coates T.D., Neumayr L.D., Treadwell M.J., Vichinsky E.P., Hulihan M.M. (2017). Emergency department utilization by Californians with sickle cell disease, 2005–2014. Pediatr. Blood Cancer.

[21] Shah N., Bhor M., Xie L., Paulose J., Yuce H. (2019). Sickle cell disease complications: Prevalence and resource utilization. PLoS ONE.

[22] Barriteau C.M., Mcnaull M.A. (2018). Sickle cell disease in the emergency department: Complications and management. Clin. Pediatr. Emerg. Med..

[23] Betz C.L., Lobo M.L., Nehring W.M., Bui K. (2013). Voices not heard: A systematic review of adolescents’ and emerging adults’ perspectives of health care transition. Nurs. Outlook.

[24] Crosby L.E., Modi A.C., Lemanek K.L., Guilfoyle S.M., Kalinyak K.A., Mitchell M.J. (2009). Perceived barriers to clinic appointments for adolescents with sickle cell disease. J. Pediatr. Hematol..

[25] Telfair J., Ehiri J.Ε., Loosier P.S., Baskin M.L. (2004). Transition to adult care for adolescents with sickle cell disease: Results of a national survey. Int. J. Adolesc. Med. Health.

[26] Hoegy D., Guilloux R., Bleyzac N., Gauthier-Vasserot A., Cannas G., Bertrand Y., Dussart C., Janoly-Dumenil A. (2022). Pediatric-Adult Care Transition: Perceptions of adolescent and young adult patients with sickle cell disease and their healthcare providers. Patient Prefer. Adherence.

[27] Oyedeji C., Strouse J.J. (2020). Improving the quality of care for adolescents and adults with sickle cell disease—It’s a long road. JAMA Netw. Open.

[28] Shukla N., Barner J.C., Lawson K.A., Rascati K.L. (2021). Age-related healthcare services utilization for the management of sickle cell disease among treated Texas Medicaid patients. J. Pharm. Health Serv. Res..

[29] Buchanan G., Vichinsky E., Krishnamurti L., Shenoy S. (2010). Severe sickle cell disease—Pathophysiology and therapy. Biol. Blood Marrow Transplant..

[30] Quinn C.T. (2013). Sickle cell disease in childhood: From newborn screening through transition to adult medical care. Pediatr. Clin. North Am..

[31] Center for Disease Control and Prevention (CDC) Preventing Sickle Cell Anemia Complications in Children. *VitalSigns*. https://www.cdc.gov/vitalsigns/sickle-cell-anemia/index.html.

[32] National Heart, Lung, and Blood Institute (2014). Evidence-Based Management of Sickle Cell Disease.

[33] Tripathi A., Jerrell J.M., Stallworth J.R. (2011). Clinical complications in severe pediatric sickle cell disease and the impact of hydroxyurea. Pediatr. Blood Cancer.

[34] Bitsko R.H., Holbrook J.R., Ghandour R.M.D., Blumberg S.J., Visser S.N.D., Perou R., Walkup J.T. (2018). Epidemiology and impact of health care provider–diagnosed anxiety and depression among US children. J. Dev. Behav. Pediatr..

[35] Pecker L.H., Darbari D.S. (2019). Psychosocial and Affective Comorbidities in Sickle Cell Disease. Neurosci Lett..

[36] Hasan S.P., Hashmi S., Alhassen M., Lawson W., Castro O. (2003). Depression in sickle cell disease. J. Natl. Med. Assoc..

[37] Jerrell J.M., Tripathi A., McIntyre R.S. (2011). Prevalence and treatment of depression in children and adolescents with sickle cell disease: A retrospective cohort study. Prim. Care Companion CNS Disord..

