# Supplementary material for: Medical Complications Among Children and Adolescents with Sickle Cell Disease in Texas Medicaid
Source: Healthcare (Basel). 2025 Sep 12;13(18):2288. doi: 10.3390/healthcare13182288 (PMC12470195; doi:10.3390/healthcare13182288)
Supplement: Supplementary file 1 [file healthcare-13-02288-s001.zip › healthcare-3835698-supplementary.pdf]

Supplementary Information

Supplementary Table S1. ICD-9 and ICD-10 Codes for Medical Complications in Patients with Sickle Cell Disease

ORGAN SYSTEM	COMPLICATIONS	ICD-9 CODES	ICD-10 CODES
**Cardio-pulmonary**	Acute chest syndrome	517.3	D57.01, D57.211, D57.411, D57.811
	Pulmonary embolism/DVT	415.1, 415.19, 416.2, 453.40	I26.09, I26.99, I27.82, I82.409
	Pulmonary hypertension	416.0, 416.8	I27.2, I27.21, I29.29, I27.8, I27.89, I27.9
**Cardiovascular**	Congestive heart failure (CHF)	428.0	I50.2, I50.2x, I50.3, I50.3x, I50.4, I50.4x, I50.9
	Cardiomegaly	429.3	I51.7
	Cardiomyopathy	425.9	I42, I42x (except I42.6; alcoholic cardiomyopathy, I42.7; cardiomyopathy due to drug and external agent
	Dysrhythmia	427.9	I49.9
**Cerebrovascular**	Acute stroke	433, 434, 436, 437.0, 437.1, 437.4, 437.5, 437.7, 437.8, 437.9	I63.0-I63.9, I60.0-I60.9, I61.0-I61.9, G45.0-G45.9
**Dermatology**	Leg ulcers	707.1x	I83.0, I83.0x, I83.0xx, I83.2, I83.2x, I83.2xx, L97, L97.x, L97.xx, L97.xxx
**Gastrointestinal**	Constipation	564.0, 564.00-564.09	K59.00-K59.09
**Genitourinary**	Priapism	607.3	N48.3, N48.30, N48.32, N48.39
**Hematological**	Aplastic episode	284.89, 284.9	D61.89, D61.9
**Hepatobiliary**	Cholelithiasis and cholecystitis	574.0-574.9, 575.0, 575.10-575.12	K80.0-K80.8, K81.0-K81.9
	Hepatic sequestration	573.8	K76.89
	Intrahepatic cholestasis	576.2	K83.1
**Musculoskeletal**	Osteonecrosis	733.40-733.49	M87.30-M87.39, M87.80-M87.89, M87.9
	Osteomyelitis	730.00-730.09	M86.00-M86.29

ORGAN SYSTEM	COMPLICATIONS	ICD-9 CODES	ICD-10 CODES
	Avascular Necrosis	733.41–733.42	M87.3, M87.3x, M87.3xx, M87.8, M87.8x, M87.8xx, M87.9
**Neurological-pain**	Vaso-occlusive crisis	282.62, 282.64, 282.69, 282.42	D57.0, D57.00, D57.21, D57.219, D57.81, D57.819, D57.41, D57.419
	Dactylitis	729.5	M79.644, M79.645, M79.646 (pain in fingers)
	Chronic pain syndrome	729.2	M54.1, M54.1x, M79.2
**Ophthalmologic**	Hyphema	364.41	H21.0, H21.0x
	Central retinal artery occlusion	362.3x	H34, H34.x, H34.xx, H43.xxx
	Proliferative sickle retinopathy	362.72	H36
**Pulmonary**	Asthma	493.1, 493.1x, 493.2, 493.2x, 493.9, 493.9x	J45.2, J45.2x, J45.3, J45.3x, J45.4, J45.4x, J45.5, J45.5x, J45.90, J45.90x
**Renal**	Hematuria	599.7, 599.7x	R31, R31.x, R31.xx
	Proteinuria	791.0	R80. R80.x
	Acute renal failure	584.6, 584.7, 584.9	N17.1, N17.2, N17.9
	Chronic kidney disease	584, 584.x, 585, 585.x 586	N17, N17.x, N18, N18.x, N19
	Hyposthenuria	403.xx-404.xx	
**Splenic**	Splenic sequestration	289.52	D57.02 (Hb-SS disease with splenic sequestration), D57.212 (Sickle-cell/Hb-C disease with splenic sequestration), D57.412 (Sickle-cell thalassemia with splenic sequestration)
	Hypersplenism	289.4	D73.1
**Other (Severe Infection)**	Meningitis	047.x, 320.x, 321.x	A87, A87.x, G00, G00.x, G01, G02, G03, G03.x
	Sepsis	995.91, 995.92, 038, 038.x, 038.xx	A41, A41.x, A41.xx, R65.2, R65.2x
**Other (Infection)**	Fever	780.61	R50.81
	Upper respiratory tract infections	465, 465.x	J00, J01, J01.x, J01.xx, J02, J02.x, J03, J03.x, J03.xx, J04, J04.x, J04.xx, J05, J05.x, J05.xx, J06, J06.x

ICD 9-/10-CM = International Classification of Diseases, Ninth/Tenth Revision, Clinical Modification

Supplementary Table S2. Proportion of Patients with 0, ≥1, ≥2, ≥3, or ≥4 Documented Complications for Each Age Group (N=1,555)

**Number of complications**	**Age group (years)**										
	**2-4 (N=364)**		**5-9 (N=419)**		**10-14 (N=426)**		**15-18 (N=346)**		**Total (N=1,555)**		**P-value[a]**
	N	%[b]	N	%[b]	N	%[b]	N	%[b]	N[c]	%	
No complications	65	17.86	79	18.85	74	17.37	92	26.59	310	19.94	0.0055*
≥ 1 complication	299	82.14	340	81.15	352	82.63	254	73.41	1245	80.06	0.0055*
≥ 2 complications	254	69.78	267	63.72	290	68.08	198	57.23	1009	64.89	0.0019*
≥ 3 complications	207	56.87	216	51.55	244	57.28	144	41.62	811	52.15	<0.0001*
≥ 4 complications	162	44.51	168	40.10	204	47.89	93	26.88	627	40.32	<0.0001*

[a]p-value from individual Chi-square tests for each row

*denotes significant p-values (<0.05) for chi-square tests

Supplementary Table S3. Proportion of Patients with Various Types of Documented Organ-System Complications by Age Group (N=1,555)

Organ system	Age group (years)										
	2-4 (N=364)		5-9 (N=419)		10-14 (N=426)		15-18 (N=346)		Total (N=1,555)		P-value
	N	%	N	%	N	%	N	%	N	%	
Cardiovascular	68	18.68	67	15.99	86	20.19	30	8.67	251	16.14	<0.0001*
Cardio-pulmonary	117	32.14	119	28.40	142	33.33	67	19.36	445	28.62	<0.0001*
Cerebrovascular	34	9.34	36	8.59	28	6.57	13	3.76	111	7.14	0.0172
Dermatological	8	2.20	10	2.39	11	2.58	4	1.16	33	2.12	0.544
Gastrointestinal	109	29.95	118	28.16	127	29.81	71	20.52	425	27.33	0.0129
Genitourinary	21	5.77	11	2.63	18	4.23	8	2.31	58	3.73	0.0488
Hematological	4	1.10	9	2.15	7	1.64	6	1.73	26	1.67	0.7256
Hepatobiliary	38	10.44	32	7.64	38	8.92	16	4.62	124	7.97	0.0308
Musculoskeletal	43	11.81	47	11.22	54	12.68	31	8.96	175	11.25	0.4234
Neurological (pain)	258	70.88	274	65.39	289	67.84	194	56.07	1015	65.27	0.0002*
Ophthalmologic	22	6.04	16	3.82	11	2.58	3	0.87	52	3.34	0.0012*
Pulmonary	106	29.12	114	27.21	116	27.23	61	17.63	397	25.53	0.0017*
Renal	71	19.51	78	18.62	74	17.37	32	9.25	255	16.40	0.0006*
Splenic	25	6.87	23	5.49	30	7.04	19	5.49	97	6.24	0.6939
Other (Infection)	156	42.86	173	41.29	203	47.65	111	32.08	643	41.35	0.0002*
Other (Severe Infection)	74	20.33	68	16.23	58	13.62	18	5.20	218	14.02	<0.0001*

p-value significance level = 0.0031 (i.e., 0.05/16) to control for Type I error rate
*denotes significant p-values for chi-square tests

Supplementary Table S4. Logistic Regression Analysis Examining the Likelihood of Experiencing Salient Documented Medical Complications by Age Group

	Overall				Vaso-occlusive Crises				Respiratory Infections				Acute Chest Syndrome			
	OR	95% CI		P-value	OR	95% CI		P-value	OR	95% CI		P-value	OR	95% CI		P-value
**Age group 5-9 vs 2-4**	0.886	0.626	1.254	0.4939	0.787	0.577	1.074	0.1316	0.995	0.735	1.349	0.9767	0.893	0.641	1.245	0.5046
**Age group 10-14 vs 2-4**	0.983	0.689	1.401	0.9227	0.835	0.610	1.143	0.2606	1.245	0.923	1.680	0.1518	0.937	0.672	1.304	0.6982
**Age group 15-18 vs 2-4**	0.691	0.483	0.991	0.0442	0.660	0.475	0.916	0.0130	0.567	0.397	0.809	0.0018	0.587	0.395	0.871	0.0081
**Female vs male**	1.008	0.784	1.296	0.9517	0.972	0.775	1.219	0.8065	1.044	0.832	1.310	0.7119	0.922	0.716	1.187	0.5277
**Resource availability Yes vs No**	1.112	0.826	1.497	0.4857	1.014	0.772	1.331	0.9220	1.246	0.934	1.662	0.1341	0.962	0.704	1.314	0.8065
**Hydroxyurea use Yes vs No**	10.833	5.292	22.173	<0.0001	7.073	4.452	11.236	<0.0001	1.944	1.467	2.578	<0.0001	4.686	3.507	6.261	<0.0001
**Mental health comorbidities Yes vs No**	1.251	0.960	1.632	0.0975	1.524	1.201	1.932	0.0005	1.022	0.810	1.290	0.8555	1.560	1.214	2.007	0.0005

Abbreviations: OR=Odds ratio; CI=Confidence Intervals
Green=significant p-values (<0.05) with odds ratios <1
Orange= significant p-values (<0.05) with odds ratios >1
